# Development and validation of an interpretable machine learning model—Predicting mild cognitive impairment in a high-risk stroke population

**DOI:** 10.3389/fnagi.2023.1180351

**Published:** 2023-06-15

**Authors:** Feng-Juan Yan, Xie-Hui Chen, Xiao-Qing Quan, Li-Li Wang, Xin-Yi Wei, Jia-Liang Zhu

**Affiliations:** ^1^Department of Geriatrics, Shenzhen Longhua District Central Hospital, Shenzhen, Guangdong, China; ^2^Department of Cardiology, Affiliated Hospital of Shandong University of Traditional Chinese Medicine, Jinan, Shandong, China; ^3^Department of Cardiology, The Third Hospital of Jinan, Jinan, Shandong, China; ^4^The First Affiliated Hospital of Jinan University, Guangzhou, Guangdong, China

**Keywords:** mild cognitive impairment, machine learning, Boruta algorithm, high-risk stroke population, prediction model

## Abstract

**Background:**

Mild cognitive impairment (MCI) is considered a preclinical stage of Alzheimer’s disease (AD). People with MCI have a higher risk of developing dementia than healthy people. As one of the risk factors for MCI, stroke has been actively treated and intervened. Therefore, selecting the high-risk population of stroke as the research object and discovering the risk factors of MCI as early as possible can prevent the occurrence of MCI more effectively.

**Methods:**

The Boruta algorithm was used to screen variables, and eight machine learning models were established and evaluated. The best performing models were used to assess variable importance and build an online risk calculator. Shapley additive explanation is used to explain the model.

**Results:**

A total of 199 patients were included in the study, 99 of whom were male. Transient ischemic attack (TIA), homocysteine, education, hematocrit (HCT), diabetes, hemoglobin, red blood cells (RBC), hypertension, prothrombin time (PT) were selected by Boruta algorithm. Logistic regression (AUC = 0.8595) was the best model for predicting MCI in high-risk groups of stroke, followed by elastic network (ENET) (AUC = 0.8312), multilayer perceptron (MLP) (AUC = 0.7908), extreme gradient boosting (XGBoost) (AUC = 0.7691), and support vector machine (SVM) (AUC = 0.7527), random forest (RF) (AUC = 0.7451), K-nearest neighbors (KNN) (AUC = 0.7380), decision tree (DT) (AUC = 0.6972). The importance of variables suggests that TIA, diabetes, education, and hypertension are the top four variables of importance.

**Conclusion:**

Transient ischemic attack (TIA), diabetes, education, and hypertension are the most important risk factors for MCI in high-risk groups of stroke, and early intervention should be performed to reduce the occurrence of MCI.

## Introduction

Cognitive dysfunction generally refers to various degrees of impairment of sensation, perception, attention, memory and other processes caused by various reasons, affecting the content of consciousness rather than the level of consciousness, including mild cognitive impairment (MCI) and dementia (Tangalos and Petersen, 2018). MCI is a transitional state between normal aging and early dementia, and is considered a preclinical stage of Alzheimer’s disease (AD), which provides a “window of opportunity” for the prevention and treatment of dementia (Petersen, 2016). Some studies (Petersen et al., 2001) have found that MCI is bidirectionally transformable, and cognitive function at this stage is reversible and can be transformed into a normal cognitive state. Early detection and reasonable intervention measures can effectively delay the formation of dementia. Previous studies have shown that age, genetic characteristics, lower educational attainment, and various clinical features are risk factors for the development of dementia (Bowler, 2005; Vanhanen et al., 2006; Razay et al., 2007; Raffaitin et al., 2009; Solfrizzi et al., 2010). A large number of studies have shown that the risk of conversion to dementia in MCI patients is much higher than that of the healthy elderly population (Jia et al., 2020). An epidemiological survey showed that the proportion of MCI among community-dwelling elderly people over 71 years old was about 21% (Plassman et al., 2008). The risk of progression to any form of dementia in patients with MCI is three to five times higher than in the general population (Petersen et al., 1999, 2009; Yaffe et al., 2006; Mitchell and Shiri-Feshki, 2008). Currently, many studies have shown that stroke and vascular risk factors (e.g., hypertension, smoking, obesity) contribute to the development of cognitive impairment and dementia (Sahathevan et al., 2012). Brain tissue may be damaged in stroke patients, and the risk of MCI is higher than that of healthy people. Therefore, we believe that identifying the occurrence of MCI in stroke patients as early as possible can reduce the incidence of MCI more effectively. As an early stage of cognitive impairment, the occurrence and development of MCI can be prevented by controlling risk factors.

There are many studies on specific disease groups with cognitive impairment (such as cerebral infarction, diabetes), but less research on cognitive impairment in stroke high-risk groups. In this study, the mini-mental state examination (MMSE) and the Montreal Cognitive Assessment (MoCA) were used to evaluate the cognitive function status of stroke high-risk groups (Kang et al., 2018).

## Materials and methods

### Data source

We collected data on 199 patients from a population at high-risk of stroke from three community health centers of Shenzhen Longhua District Central Hospital from June 2021 to June 2022 as the research objects.

### Inclusion and exclusion criteria

Inclusion criteria: (1) patients with 3 or more stroke risk factors among hypertension, diabetes, atrial fibrillation, valvular heart disease, dyslipidemia, smoking history, obesity, lack of exercise, and family history of stroke, or with transient ischemic attack (TIA); (2) people aged 60∼80; (3) those who signed the informed consent form.

Exclusion criteria: (1) patients with acute stroke who have been discharged from hospital for less than 6 months, or who have severe heart, liver, lung, kidney and other life-threatening conditions or who cannot cooperate with investigation and evaluation; (2) patients with a definite diagnosis of dementia which affect their daily and self-care ability.

### Research variable

A total of 46 variables were included in this study. The variables include basic information of patients, vital signs, laboratory tests, complications, and medication history, with the number of variables for each category being 4, 6, 31, 4, and 1, respectively. Basic information included education, age, sex, and smoking history, Illiteracy, primary school and junior high school education are defined as lower education level; high school education and above are defined as higher education level. Vital signs include systolic blood pressure, diastolic blood pressure, temperature, heart rate, respiratory rate, body mass index. Laboratory items include white blood cells, red blood cells, hemoglobin, platelets, hematocrit, mean corpuscular volume, mean corpuscular hemoglobin volume, mean corpuscular hemoglobin concentration, red blood cell distribution width, triglycerides, total cholesterol, HDL cholesterol, LDL cholesterol, serum glucose, serum sodium, serum chloride, serum potassium, serum calcium, serum bicarbonate, serum creatinine, serum uric acid, serum albumin, total bilirubin, alanine aminotransferase, aspartate aminotransferase, creatine kinase myocardial band, homocysteine, prothrombin time, activated partial thromboplastin time, international normalized ratio, D-dimer. Comorbidities include TIA, hypertension, diabetes, coronary heart disease. Medication history was defined as taking one of aspirin, clopidogrel, or ticagrelor.

### Statistical analysis

The Boruta algorithm is currently a very popular feature screening method (Lei et al., 2021). We integrated the filtered variables into the machine learning model. In this study, we aimed to compare eight machine learning methods to build the model and select the model with the best performance for model interpretation. In order to improve the efficiency of use and clinical usability, we developed an online risk calculator using the best model, which can effectively help doctors identify the risk of MCI in stroke high-risk groups.

The Boruta algorithm is used for feature importance selection, the core of which is based on shadow features. We randomly scramble each feature, these scrambled features are called shadow features, and take the mean of feature importance before selection. The features most associated with the dependent variable are then included in the model. Eight machine learning algorithms are used to build the model, which are logistic regression (LR), decision tree (DT), K-nearest neighbors (KNN), random forest (RF), extreme gradient boosting (XGBoost), elastic network (ENET), support vector machine (SVM), multilayer perceptron (MLP). Before the algorithm starts, we set the hyperparameters (Supplementary Table 1) to improve the performance and effect of the machine learning model. The specific hyperparameters can be seen in the Supplementary material. The data of 199 patients were randomly divided into training set (70%) and testing set (30%) according to the ratio of 7:3. In order to assess the robustness of the model, we employ fivefold cross-validation on the training set and testing set. On the training set, eight machine learning algorithms were used to build the model, and the testing set was used to test the effectiveness of the model. The performance metrics of the eight machine learning models are represented by parallel line graphs. In addition, a calibration curve was used to assess the agreement between observed and predicted probabilities, and a decision curve (DCA) was used to assess clinical validity. We defined the model with the maximum value of the area under the curve (AUC) of the receiver operating characteristic curve (ROC) as the best model.

Variable importance is used to show the importance of each feature to the model output, and select the top four variables for discussion. In addition, shapley additive explanation (SHAP) is used for model visualization. Firstly, the SHAP summary plot was used to illustrate the effects of the features attributed to the model. Secondly, partial dependence plots were used to analyze the effect of a feature on the outcome. Finally, single-sample SHAP is used to demonstrate the impact of features on the outcome of a single forecast sample. If the SHAP value assigned to each feature in the forecast sample is greater than 0, it is positively correlated with the outcome, and if it is less than 0, it is negatively correlated with the outcome. Continuous variables were represented using medians and quartiles, compared by using the Wilcoxon rank sum test. Categorical variables were expressed using frequencies and percentages, and chi-square tests or Fisher’s exact probability method were used for comparisons.

All analyzes were performed using R software (version 4.2.0), and two-sided *P*-values < 0.05 were considered statistically significant. The used R packages include tidymodels, Boruta, rpart.plot, NeuralNetTools, pROC, PredictABEL, iml, fastshap, gtsummary, Table 1, dplyr.

**TABLE 1:** Descriptive characteristics of overall participants.

Variables	Total (*n* = 199)	Control group (*n* = 111)	Incidence group (*n* = 88)	*P*-value
Age (year)	68 (64, 72)	67 (63, 72)	68 (65, 73)	0.062
SBP (mmHg)	134 (124, 152)	131 (122, 148)	136 (126, 155)	0.048
DBP (mmHg)	81 (75, 90)	81 (74, 90)	82 (76, 89)	0.445
Body temperature (°C)	36.50 (36.40, 36.60)	36.50 (36.35, 36.60)	36.50 (36.40, 36.60)	0.759
Heart rate (beats/min)	77 (67, 87)	77 (67, 87)	77 (68, 87)	0.797
Respiratory rate (beats/min)	20 (19, 20)	20 (20, 20)	20 (19, 20)	0.777
BMI	23.96 (21.95, 25.87)	24.13 (22.03, 26.51)	23.76 (21.73, 25.64)	0.363
WBC (10^9^/L)	6.60 (5.28, 7.99)	6.60 (5.18, 8.13)	6.57 (5.40, 7.92)	0.624
RBC (10^12^/L)	4.44 (4.15, 4.88)	4.43 (4.17, 4.88)	4.44 (4.06, 4.88)	0.734
Hemoglobin (g/L)	133 (122, 142)	135 (125, 142)	132 (120, 142)	0.291
PLT (10^9^/L)	220 (176, 262)	213 (174, 258)	225 (177, 264)	0.639
HCT (%)	40.1 (36.8, 42.8)	40.6 (37.3, 42.8)	39.5 (34.8, 42.4)	0.094
MCV (fl)	89 (86, 92)	90 (86, 92)	88 (86, 91)	0.143
MCH (pg)	30.10 (28.80, 30.90)	30.10 (28.95, 30.85)	30.05 (28.60, 30.92)	0.868
MCHC (g/L)	332 (325, 344)	333 (325, 343)	332 (324, 345)	0.599
RDW (%)	13.10 (12.50, 13.70)	12.90 (12.40, 13.75)	13.20 (12.60, 13.70)	0.248
Triglycerides (mmol/L)	1.41 (0.99, 1.96)	1.34 (0.94, 2.00)	1.49 (1.14, 1.92)	0.249
Total cholesterol (mmol/L)	4.36 (3.37, 5.16)	4.38 (3.33, 5.20)	4.35 (3.38, 5.15)	0.818
HDL (mmol/L)	1.14 (0.99, 1.35)	1.18 (0.99, 1.35)	1.12 (1.00, 1.34)	0.798
LDL (mmol/L)	2.36 (1.74, 3.02)	2.35 (1.74, 3.11)	2.38 (1.74, 2.94)	0.756
Serum glucose (mmol/L)	5.99 (5.25, 7.81)	5.87 (5.14, 7.67)	6.23 (5.38, 8.25)	0.091
Serum sodium (mmol/L)	141.70 (139.80, 143.15)	141.80 (140.00, 143.10)	141.20 (139.57, 143.12)	0.277
Chloride (mmol/L)	105.0 (102.2, 106.7)	105.0 (102.7, 106.3)	105.0 (102.2, 107.0)	0.859
Serum potassium (mmol/L)	4.09 (3.84, 4.31)	4.04 (3.85, 4.28)	4.12 (3.83, 4.32)	0.286
Serum calcium (mmol/L)	2.29 (2.23, 2.36)	2.28 (2.22, 2.37)	2.30 (2.24, 2.35)	0.559
Bicarbonate (mmol/L)	24.00 (22.30, 26.20)	24.10 (22.40, 26.55)	23.95 (22.08, 25.83)	0.171
Serum creatinine (umoI/L)	74 (62, 90)	71 (60, 88)	78 (65, 94)	0.044
Uric acid (mg/dL)	340 (296, 406)	327 (274, 394)	364 (316, 429)	0.011
Serum albumin (g/L)	42.1 (39.9, 45.1)	42.2 (40.0, 45.5)	42.0 (39.9, 44.5)	0.254
Total bilirubin (umol/L)	11.1 (7.8, 15.1)	11.3 (7.8, 14.5)	10.8 (8.1, 15.1)	0.973
ALT (U/L)	19 (14, 25)	20 (14, 25)	18 (14, 23)	0.327
AST (U/L)	23 (20, 27)	24 (20, 28)	23 (20, 26)	0.358
CKMB (U/L)	10 (1, 15)	11 (1, 15)	10 (1, 15)	0.760
Homocysteine (umol/L)	12.0 (9.4, 15.8)	11.2 (9.0, 14.9)	13.4 (10.3, 17.3)	0.008
PT (s)	11.70 (11.00, 12.40)	11.80 (11.20, 12.50)	11.55 (10.80, 12.22)	0.129
APTT (s)	25.6 (23.1, 28.0)	25.8 (23.3, 28.6)	25.4 (23.0, 27.4)	0.375
INR	1.03 (0.96, 1.08)	1.03 (0.97, 1.09)	1.03 (0.95, 1.07)	0.290
D-dimer (mg/L)	0.39 (0.24, 0.62)	0.38 (0.21, 0.56)	0.44 (0.26, 0.69)	0.067
Education (*n*, %)				0.001
1	152 (76%)	75 (68%)	77 (88%)	
2	47 (24%)	36 (32%)	11 (12%)	
Sex (*n*, %)				0.727
Male	99 (50%)	54 (49%)	45 (51%)	
Female	100 (50%)	57 (51%)	43 (49%)	
Smoke (*n*, %)				0.342
No	149 (75%)	86 (77%)	63 (72%)	
Yes	50 (25%)	25 (23%)	25 (28%)	
TIA (*n*, %)				< 0.001
No	135 (68%)	91 (82%)	44 (50%)	
Yes	64 (32%)	20 (18%)	44 (50%)	
Hypertension (*n*, %)				0.015
No	35 (18%)	26 (23%)	9 (10%)	
Yes	164 (82%)	85 (77%)	79 (90%)	
Diabetes (*n*, %)				0.017
No	70 (35%)	47 (42%)	23 (26%)	
Yes	129 (65%)	64 (58%)	65 (74%)	
CAD (*n*, %)				0.976
No	97 (49%)	54 (49%)	43 (49%)	
Yes	102 (51%)	57 (51%)	45 (51%)	
Drug (*n*, %)				0.135
No	55 (28%)	26 (23%)	29 (33%)	
Yes	144 (72%)	85 (77%)	59 (67%)	

Education: 1, lower education level; 2, higher education level.

## Results

The baseline characteristics of the patients are presented in Table 1. In this study, a total of 199 patients with high-risk of stroke were included, with a median age of 68 years (interquartile range, 64–72 years old), of whom 88 (44.2%) had MCI, and 99 (49.7%) were male. The systolic blood pressure, creatinine, uric acid and homocysteine in the disease group were higher than those in the control group, and the difference was statistically significant. In the higher education group, the incidence of MCI was lower than that in the lower education group, and the difference was significant. Among people with diabetes, hypertension, and TIA, the number of people with MCI was more than those without the above three diseases, and the difference was significant.

### Model building and verification

**FIGURE 1:**
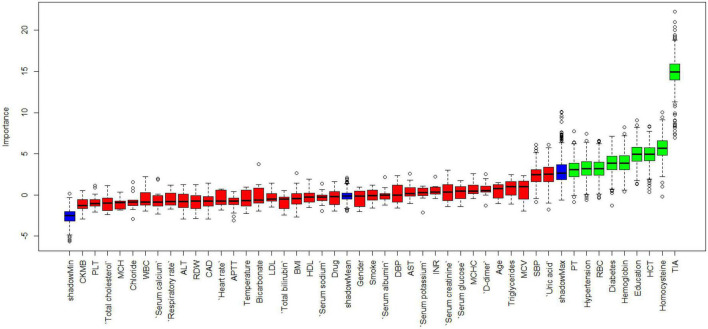
Feature selection based on Boruta algorithm.

**FIGURE 2:**
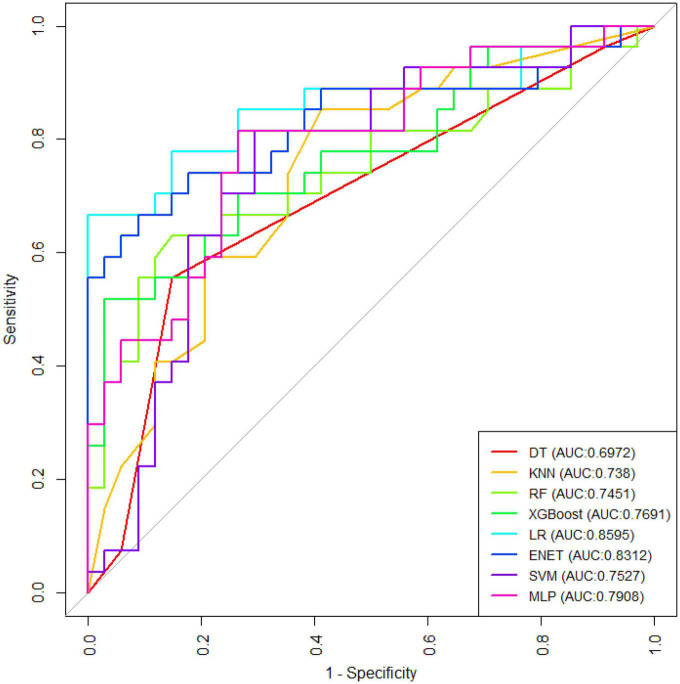
Receiver operating characteristic curve of the eight models.

**FIGURE 3:**
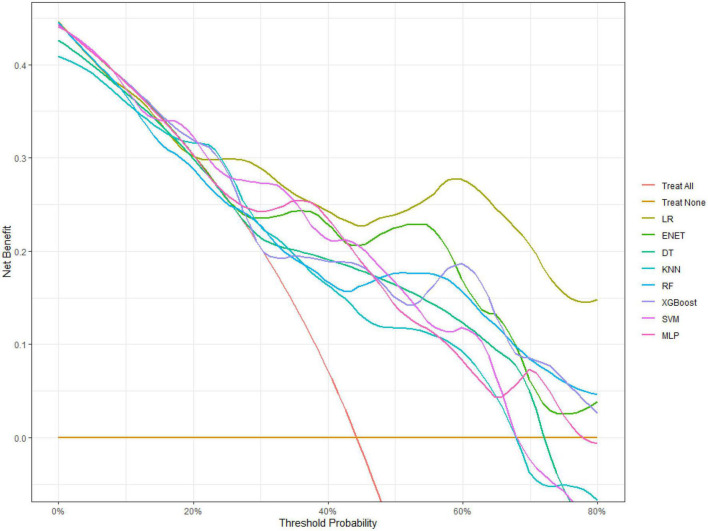
Decision curve analysis of eight types of machine learning.

**FIGURE 4:**
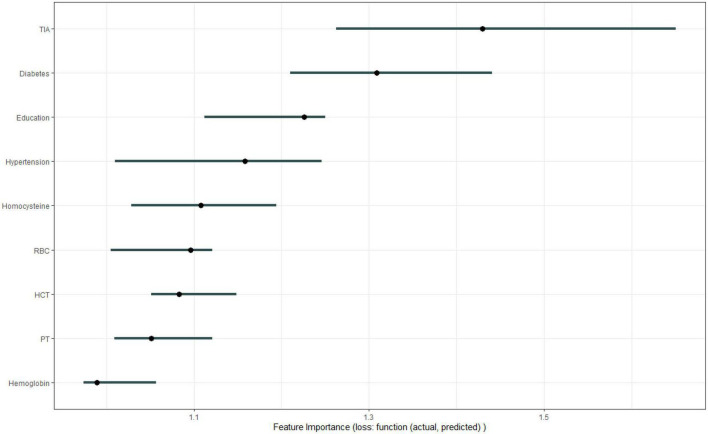
Variable importance.

**FIGURE 5:**
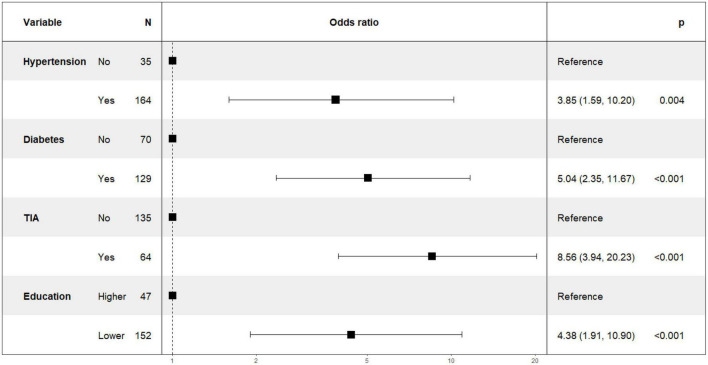
Odds ratios and 95% confidence intervals for hypertension, diabetes, TIA, education.

Figure 1 shows the results of feature selection based on the Boruta algorithm. Sorted according to the Z score value, the green ones are considered acceptable variables, a total of 9 variables, namely TIA, homocysteine, education, HCT, diabetes, hemoglobin, RBC, hypertension, PT. Using the above nine variables, eight machine learning models were established to predict the risk of MCI in stroke high-risk groups. Figure 2 shows the ROC curve of each model, and the model effect is expressed by AUC value. Logistic regression (AUC = 0.8595) was the best model for predicting MCI in high-risk groups of stroke, followed by ENET (AUC = 0.8312), MLP (AUC = 0.7908), XGBoost (AUC = 0.7691), and SVM (AUC = 0.7527), RF (AUC = 0.7451), KNN (AUC = 0.7380), DT (AUC = 0.6972). Supplementary Figure 1 shows other indicators of each model, in which the accuracy of logistic regression is 0.770, the sensitivity is 0.778, the specificity is 0.765, and the recall is 0.778. Supplementary Figure 2 shows the calibration curves of each model. The calibration curve of the logistic regression model did not deviate significantly from the reference line, indicating that it has good predictive performance. According to the DCA curve (Figure 3), logistic regression showed a greater net benefit, indicating good clinical validity of the logistic regression model. Figure 4 shows the importance of variables, among which the top four are TIA, diabetes, education level, and hypertension. The forest plot (Figure 5) shows the odds ratio (OR) and 95% confidence interval (95% CI) of the top 4 variables of importance. The (OR, 95% CI) of patients with hypertension, diabetes and TIA were 3.85 (1.59–10.2), 5.04 (2.35–11.67), 8.56 (3.94–20.23), respectively, and the difference was significant. The (OR, 95% CI) of lower education level was 4.38 (1.91–10.9), and the difference was significant. In order to improve the speed of operation and clinical utility, the most efficient logistic regression model was used to develop an online risk calculation^1^ to assess the risk of MCI in high-risk stroke populations.

### Model interpretation

**FIGURE 6:**
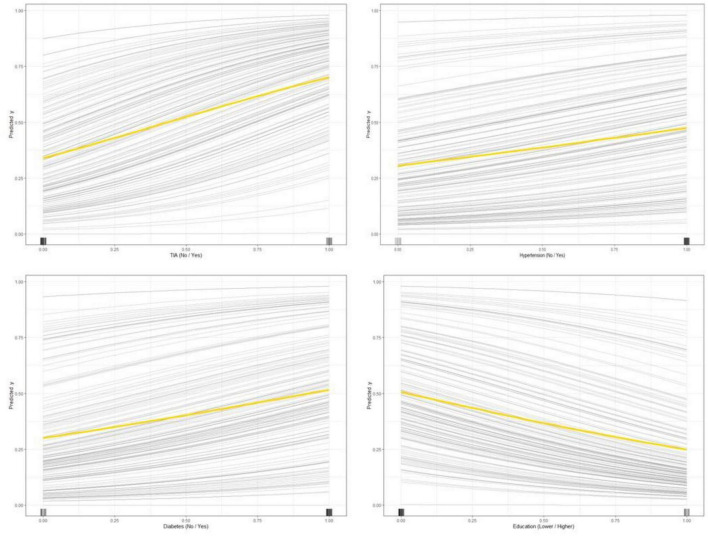
Partial dependence plot of features (TIA, diabetes, education level, hypertension).

**FIGURE 7:**
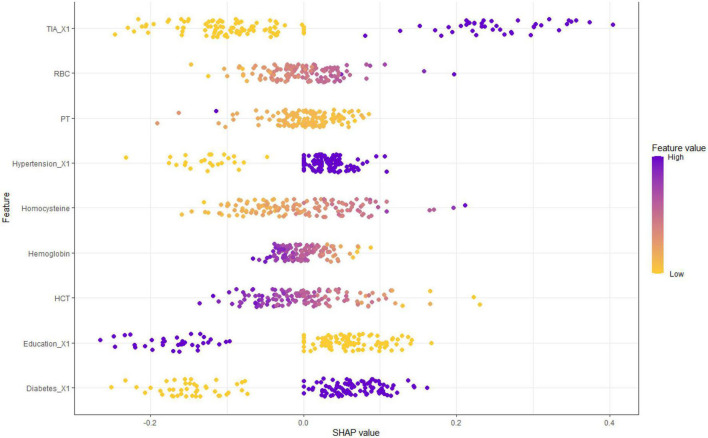
SHAP value according to the feature of MCI in high-risk groups of stroke.

**FIGURE 8:**
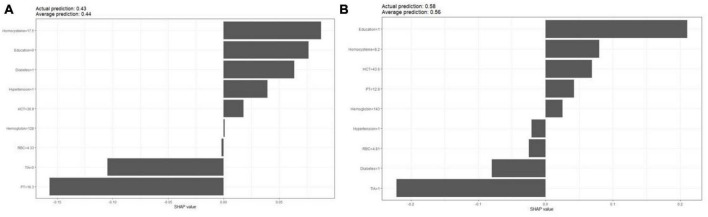
SHAP values for each feature of a single sample. **(A)** MIC occurs; **(B)** MIC does not occur.

The partial dependence plot (Figure 6) shows the effect of the top four most important variables on the outcome in the logistic regression model. Among high-risk groups of stroke, TIA, diabetes, and hypertension were positively correlated with the occurrence of MCI, while higher education was negatively correlated with the risk of developing MCI. The SHAP summary plot (Figure 7) shows the impact of each variable on the outcome in the logistic regression model, including positive and negative. Each point in Figure 7 represents the SHAP value of each sample. In this study, for continuous variables, purple indicates that the value is larger, and yellow indicates that the value is smaller. The more dispersed the points, the greater the impact of the variable on the outcome of the model; for binary variables, purple represents occurred, and yellow represents not occurred. Figure 7 shows that patients with TIA, diabetes and hypertension have a positive SHAP value, which is more conducive to the occurrence of MCI. Higher education has a lower SHAP value, indicating that higher education prevents the occurrence of MCI. Figure 8A shows the single-sample predictions of MCI, with TIA, diabetes, and hypertension contributing to the occurrence of the disease, while higher education was protective. Figure 8B shows the prediction of a single sample without MCI, high education and no TIA are beneficial to prevent the occurrence of MCI, while diabetes and hypertension promote the occurrence of MCI.

## Discussion

In this retrospective cohort study, Boruta algorithm was used to screen 46 variables, and a machine learning model was developed and validated to predict the risk of MCI in stroke high-risk population. Machine learning models can be used to realize early dynamic monitoring, which can save clinicians’ time (Li et al., 2020). Artificial intelligence and machine learning are gaining popularity in clinical research, such as assessing patient outcomes after surgery (Voglis et al., 2020), predicting hypotension (Kendale et al., 2018), and depth of anesthesia (Lee et al., 2018).

Firstly, the Boruta algorithm shuffles each feature value of the feature matrix, and splices the shuffled features (shadow features) with the original features (real features) to form a new feature matrix. Secondly, calculate the Z-score of the real features and the shadow features. Find the largest Z-score in the shadow features and define it as Z-max. Mark a real feature with a Z-score greater than Z-max as “important.” Set all other real features whose Z-score is significantly smaller than Z-max as “unimportant” and permanently remove them from the feature set. Repeat the above steps until all features are marked as “important” or “unimportant” (Kursa and Rudnicki, 2010). Finally, we obtain the eigenvalues of the three color channels. The red area represents the rejection zone, and features in this area are considered noise and can be discarded. The blue area is the hesitation zone, which poses difficulty for Boruta in selecting the features. The green region is considered the acceptable area, where features are generally considered predictive and can be kept. In this study, we included variables in the green area into the model. In the machine learning modeling process, we use grid search and fivefold cross-validation to find the hyperparameters of the model. The training cohort is randomly divided into 5 subsets, one of which is selected as the validation data set, and the other four are used as the training data set, and five iterations are performed to obtain a reliable and stable model. Our DCA suggests that the logistic regression model has good clinical utility.

As one of the machine learning algorithms, logistic regression has been compared with other machine learning algorithms in previous studies, and it has been shown that other machine learning algorithms do not necessarily perform better than logistic regression. Logistic regression provides odds ratios that are easily interpreted. The importance output of machine learning for individual predictors is not very informative. Our research also shows that logistic regression models perform best (Kuhle et al., 2018). Logistic regression models were interpreted using SHAP. Variable importance found that TIA, diabetes, education level, and hypertension were the top four variables with the greatest influence on predicting MCI. The odds ratios of the above four variables were calculated using a logistic regression model, and the results were presented in a forest plot. We used the SHAP force to predict individual and overall MCI in the high-risk group of stroke in the logistic regression model, and the results showed that diabetes, TIA, hypertension, and lower education level promoted the occurrence of MCI.

Studies have shown that about one-third of stroke patients develop MCI (Sachdev et al., 2006), and some patients may recover over time (Desmond et al., 1996), but the overall cognitive function shows a downward trend, which is due to stroke Patients are at increased risk of cerebrovascular disease progression (Wentzel et al., 2001; Aharon-Peretz et al., 2002; Nyenhuis et al., 2002; Tham et al., 2002; Srikanth et al., 2004; del Ser et al., 2005). A previous study showed that transient cognitive impairment was common and most patients were asymptomatic when evaluated within 7 days of TIA (Pendlebury et al., 2011). Meanwhile, some TIA patients developed MCI after the first day (Pendlebury et al., 2011). Our results show that TIA is an important risk factor for MCI in stroke high-risk groups, which is consistent with previous research results.

Hypertension has been confirmed to be closely related to cerebral small vessel lesions such as white matter lesion (WML), lacunar infarction, or cerebral microbleeds (Viswanathan et al., 2009), and these diseases also play an important role in the process of dementia (Debette and Markus, 2010). In addition, hypertension can easily lead to atherosclerosis and tortuosity of small blood vessels in the cerebrovascular system, and pathological changes in these blood vessels can lead to vascular stenosis and decreased perfusion. This hypoperfusion promotes diffuse ischemic changes in the deep white matter, leading to vascular cognitive impairment (O’Brien et al., 2003). The study by Skoog et al. (1996) found that elevated blood pressure at the age of 70 was associated with the development of dementia 10–15 years later, suggesting that previous elevated blood pressure may lead to the development of dementia through WML. Our study also showed that among high-risk groups of stroke, compared with those without hypertension, the risk of MCI in patients with hypertension increased by 3.85 times, and the difference was significant (*P*-value < 0.05).

The results of our study showed that the risk of MCI increased by 5.04 times in patients with diabetes comorbidities among the high-risk groups of stroke. Meta-analyses of an increasing number of observational studies have shown that diabetes has a large adverse effect on cognitive function (Cheng et al., 2012; Sadanand et al., 2016; Zhang et al., 2017). A study by Zhou et al. (2010) showed that in diabetic patients, the cognitive subdomains served by the frontotemporal cortex are affected, leading to a decline in cognitive functions such as memory and processing ability. In addition, in the non-demented population, the relative risk of MCI in diabetic patients was 1.49 (Xue et al., 2019), which is consistent with the results of Cheng’s meta-analysis. The results of a prospective study in 2019 showed that prediabetes was associated with accelerated decline in cognitive function and was associated with smaller overall brain volume, especially lower white matter volume (Marseglia et al., 2019). Our study showed that the risk of MCI increased 4.38 times with lower educational level. Studies have shown that a higher education level can effectively delay the decline of individual cognitive function (Vadikolias et al., 2012). At the same time, for people with higher education, it is more conducive to understand the assessment scale and implement it perfectly, which may also be one of the reasons for the lower incidence of MCI. At the same time, when MCI is diagnosed, relevant treatment should be given as soon as possible. Studies have shown that reasonable exercise, good sleep, stress management, mental exercise, optimization of gut and oral microbiome, optimization of nutritional support, reduction of inflammation, and neutralization of free radicals will promote the reversal of MCI. Therefore, early recognition of MCI and early intervention are crucial for the treatment of MCI (Rao et al., 2023).

This study compared eight machine learning models for the first time to comprehensively analyze, predict the risk of MCI in high-risk stroke groups, and identify the most important risk factors, which are the highlights of this study. This can effectively enable patients to intervene on the risk factors of MCI before the onset of stroke, which is more conducive to preventing the occurrence of MCI. Our study also has some limitations. Firstly, the included sample size is not large, and the established model may not be effective enough. Secondly, this is a single-center retrospective study, and we hope that in future studies, a multi-center population can be included as an external validation to obtain more information.

## Conclusion

Transient ischemic attack (TIA), diabetes, education, and hypertension are the most important risk factors for MCI in high-risk population of stroke, and early intervention should be performed to reduce the occurrence of MCI.

## Data availability statement

The original contributions presented in this study are included in the article/Supplementary material, further inquiries can be directed to the corresponding author.

## Ethics statement

The studies involving human participants were reviewed and approved by the Medical Ethics Committee of Shenzhen Longhua District Central Hospital. The patients provided their written informed consent to participate.

## Author contributions

J-LZ contributed to the study concept and study design. F-JY performed statistical analysis and data interpretation. X-HC and X-QQ performed literature research and data extraction. L-LW and X-YW were responsible for the quality control of data and algorithms. All authors contributed to writing of the manuscript and approved the final version.

## Footnotes

1
https://shrpmci.shinyapps.io/shrpmciprediction/
