# Supplementary material for: Development and validation of an interpretable machine learning model—Predicting mild cognitive impairment in a high-risk stroke population
Source: Front Aging Neurosci. 2023 Jun 15;15:1180351. doi: 10.3389/fnagi.2023.1180351 (PMC10308219; doi:10.3389/fnagi.2023.1180351)
Supplement: Supplementary file 1 [file Table_1.docx]

Table S1

Machine Learning Algorithm Hyperparameters

**Decision tree:**

tree_depth = tune(),

min_n = tune(),

cost_complexity = tune()

**Random forest:**

mtry = tune(),

trees = tune(),

min_n = tune()

**Xgboost:**

mtry = tune(),

trees = 1000,

min_n = tune(),

tree_depth = tune(),

learn_rate = tune(),

loss_reduction = tune(),

sample_size = tune(),

stop_iter = 25

**ENET:**

mixture = tune(),

penalty = tune()

**SVM:**

cost = tune(),

rbf_sigma = tune()

**MLP:**

hidden_units = tune(),

penalty = tune(),

epochs = tune()

**KNN:**

neighbors = tune(),

weight_func = "optimal",

dist_power = 2
