# Supplementary material for: Development and validation of an interpretable machine learning model—Predicting mild cognitive impairment in a high-risk stroke population
Source: Front Aging Neurosci. 2023 Jun 15;15:1180351. doi: 10.3389/fnagi.2023.1180351 (PMC10308219; doi:10.3389/fnagi.2023.1180351)
Supplement: Supplementary file 2 [file Data_Sheet_1.docx]

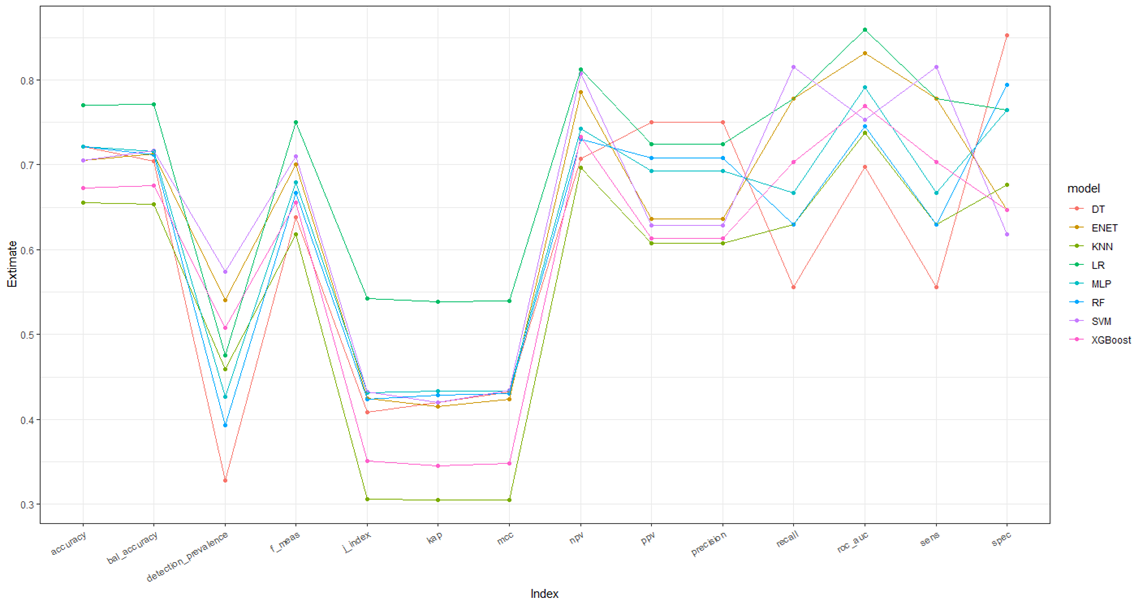


Figure S1: Metrics for eight machine learning models


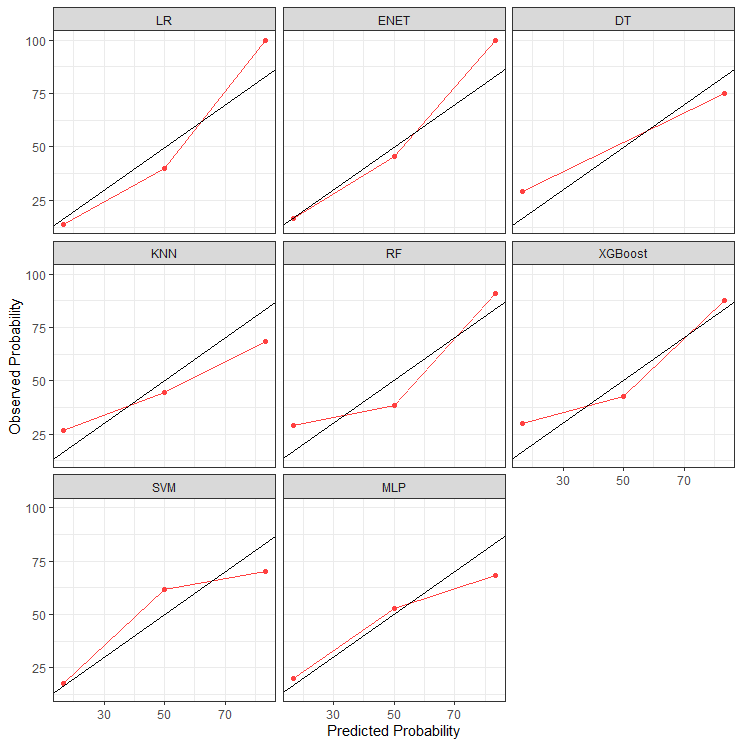


Figure S2: Calibration curves of eight machine learning models
